# The association of household food insecurity and HIV infection with common mental disorders among newly diagnosed tuberculosis patients in Botswana

**DOI:** 10.1017/S1368980020004139

**Published:** 2022-04

**Authors:** Qiao Wang, Mbatshi Dima, Ari Ho-Foster, Keneilwe Molebatsi, Chawangwa Modongo, Nicola M Zetola, Sanghyuk S Shin

**Affiliations:** 1 Sue & Bill Gross School of Nursing, University of California Irvine, 106F Berk Hall, Irvine, CA 92697, USA; 2 Botswana-UPenn Partnership, Gaborone, Botswana; 3 Office of Research and Graduate Studies, Faculty of Medicine, University of Botswana, Gaborone, Botswana; 4 Perelman School of Medicine, University of Pennsylvania, PA, USA; 5 Department of Psychiatry, University of Botswana, Gaborone, Botswana

**Keywords:** HIV infection, HIV/tuberculosis co-morbidity, Mental illness, Common mental disorders, Epidemic

## Abstract

**Objective:**

To determine the association between food insecurity and HIV infection with depression and anxiety among new tuberculosis (TB) patients.

**Design:**

Our cross-sectional study assessed depression, anxiety and food insecurity with Patient Health Questionnaire (PHQ-9), Zung Anxiety Self-Assessment Scale (ZUNG) and Household Food Insecurity Access Scale, respectively. Poisson regression models with robust variance were used to examine correlates of depression (PHQ-9 ≥ 10) and anxiety (ZUNG ≥ 36).

**Setting:**

Gaborone, Botswana.

**Participants:**

Patients who were newly diagnosed with TB.

**Results:**

Between January and December 2019, we enrolled 180 TB patients from primary health clinics in Botswana. Overall, 99 (55·0 %) were HIV positive, 47 (26·1 %), 85 (47·2 %) and 69 (38·5 %) indicated depression, anxiety and moderate to severe food insecurity, respectively. After adjusting for potential confounders, food insecurity was associated with a higher prevalence of depression (adjusted prevalence ratio (aPR) = 2·30; 95 % CI 1·40, 3·78) and anxiety (aPR = 1·41; 95 % CI 1·05, 1·91). Prevalence of depression and anxiety was similar between HIV-infected and HIV-uninfected participants. Estimates remained comparable when restricted to HIV-infected participants.

**Conclusions:**

Mental disorders may be affected by food insecurity among new TB patients, regardless of HIV status.

Mental illness is a leading cause of disability, with approximately 970 million people being affected worldwide^(1)^. Compared with the general population, individuals affected by tuberculosis (TB) are significantly more likely to have poor mental health due to biological and social complexities^(2)^. Common mental illness, including depression and anxiety disorders, imposes immense costs on healthcare systems and poor quality of life on patients and can be important barriers for TB diagnosis and treatment. Moreover, depression may lead to medication nonadherence resulting in unfavourable TB treatment outcomes and ongoing community transmission^(3)^, which has been cited as the fundamental obstacle in effective TB control^(4)^. Therefore, mental health is an integral part of achieving WHO’s The End TB Strategy and eliminating the global TB epidemic^(5)^. Unfortunately, the majority of TB and mental illness occur in low- and middle-income countries^(6)^, where mental health is often overlooked due to a myriad of challenges such as lack of mental health specialists, limited treatment options, perceived stigma and other competing public health priorities^(6)^.

Meanwhile, individuals with TB often experience malnutrition^(7)^, which may be exacerbated by food insecurity. Globally, about 2 billion people suffer from moderate to severe levels of food insecurity^(8)^. According to The United States Department of Agriculture, a household is defined as food insecure if at least one member experiences insufficient or inconsistent access to nutritionally adequate and safe food^(9)^. A survey of patients with TB/HIV co-infection in multiple African cities reported that only 10 % TB patients feel they are food secure^(10)^. Food insecurity not only negatively affects general physical health but also has important ramifications for adverse mental health. For instance, studies have shown that adults with food insecurity are more likely to have anxiety disorder, depression and suicidal ideation compared with those who are food secure^(11)^.

Globally, Botswana has one of the highest TB incidence – 300 cases per 100 000 population in 2017^(12)^. About 60 % of all TB patients also have co-morbid HIV infection^(12)^, which has been suggested as a risk factor in psychiatric populations^(13,14)^. People living with HIV/AIDS may have an increased risk of developing mood disorders and clinical depressive symptoms triggered by distress associated with initial HIV diagnosis, disease progression, difficult life circumstances, hospitalisation, HIV-induced brain injury and adverse effects of antiretroviral medications^(14)^. The most recent data indicate that psychiatric morbidity in the country contributes an estimated 4·6 % of the global burden of disease^(15)^. There are 0·5 mental health outpatient facilities, 2·5 psychiatrists and 15·2 psychologists available per million population in spite of the heavy burden of psychological distress^(15)^.

For the past few decades, Botswana has been experiencing rapid urbanisation and positive economic growth with a significant decline in poverty incidence^(16)^. At the same time, many challenges remain, including food insecurity. The Food and Agricultural Organisation ( FAO) indicated that the average prevalence of severe food insecurity was 41·3 % between 2016 and 2018^(17)^. It is crucial to understand the relationship between food insecurity and mental health among the socially vulnerable TB patients because, while the expansion and integration of mental health care can be a challenging and gradual task in resource-limited settings, social support programmes, particularly food supplementation, may be relatively low intensity and straightforward for policy delivery and implementation. The objectives of our study were to examine the prevalence of depression and anxiety among newly diagnosed TB patients in Botswana and to explore the association between those conditions with food insecurity and HIV co-infection.

## Methods

### Study design, population and eligibility

We conducted a cross-sectional study and recruited participants with newly diagnosed pulmonary or extrapulmonary TB from primary healthcare clinics in Gaborone, Botswana. Participants were deemed eligible if they were at least 18 years old with a verified TB diagnosis and were recruited by our research staff at the time of receiving TB diagnosis. According to national guidelines during the study period, the first-line diagnostic test for TB was sputum smear microscopy or GeneXpert MTB/RIF for patients who experienced prolonged general pulmonary TB symptoms such as cough, fever, shortness of breath, night sweats, weight loss and haemoptysis^(18)^. Patients with indications for culture testing, such as symptomatic individuals at high risk of multidrug-resistant TB, also underwent examination by mycobacterial culture and drug susceptibility testing with the Mycobacteria Growth Indicator Tube method. A chest radiography was performed in symptomatic individuals who had negative sputum smears or negative Xpert MTB/RIF. For patients who had negative results in all testing, TB diagnosis was made by a physician based on clinical presentations and/or lymph node examinations. A rapid HIV test was conducted among participants who have not had an HIV test done before, or for those who have had a negative result in previous testing.

### Data collection

Socio-demographic data were collected via face-to-face interview and included age, gender, education, monthly income, marital status, smoking and alcohol history. We also collected clinical information that included TB symptoms, duration of the symptoms, HIV test result and HIV diagnosis date if any, antiretroviral treatment (ART) history, and results of smear microscopy, Xpert MTB/RIF, mycobacterial culture and chest X-ray. CD4 T-cell data closest to the time of enrolment were extracted and integrated from the national HIV database for HIV-infected participants.

We administered standardised questionnaires to assess the presence and severity of depressive symptoms, anxiety symptoms and household food insecurity of enrolled participants. For simplicity, we will use depression and anxiety to describe depressive symptoms and anxiety symptoms, respectively, hereinafter.

Depression was assessed by the Patient Health Questionnaire (PHQ-9), a nine-item self-report tool that screened depression as participants identified how much each item statement applied to them within the past 2 weeks^(19)^. Examples of item statements include ‘feeling down, depressed or hopeless’ and ‘little interest or pleasure in doing things’. Answer choices were scored on a Likert-type scale that ranged from ‘not at all’, ‘several days’, ‘more than half the days’, to ‘nearly every day’. PHQ-9 has been validated in multiple sub-Saharan African countries to assess depression, including Botswana^(20)^.

Anxiety was assessed by Zung Self-Rating Anxiety Scale (ZUNG), a twenty-item self-report scale that measured anxiety levels^(21)^. Similar to PHQ-9, participants indicated how much each of the twenty statements applied to them within the past several days and replies included ‘none or a little of the time’, ‘some of the time’, ‘good part of the time’ and ‘most or all of the time’. Examples of item statements include ‘I feel afraid for no reason at all’ and ‘I get upset easily or feel panicky’.

Finally, food insecurity was evaluated by the Household Food Insecurity Access Scale, a nine-item questionnaire widely used across different cultures, including Botswana^(16)^, to distinguish food-insecure households and to determine the degree of food insecurity^(22)^. Examples on this questionnaire include ‘In the past four weeks, did you worry that your home would not have enough food?’ and ‘In the past four weeks, did you or anyone in the house go to sleep at night hungry because there was not enough food?’ Answer choices were scored on a Likert-type scale that ranged from ‘never’, ‘rarely (1 or 2 times)’, ‘sometimes (3–10 times)’, to ‘often (more than 10 times)’.

All questionnaires were prepared in English and Setswana, both official languages of Botswana. Study interview was conducted in the preferred language of the participant. We restate that data collection tools (i.e. PHQ-9 and ZUNG) used in this study were suggestive of symptoms of depression and anxiety, and not used for clinical diagnoses. All participants with PHQ-9 scores indicative of depression and suicidal ideation were provided with appropriate psychiatric referrals.

### Statistical analysis

The outcomes of interest for this study were the prevalence of depression and anxiety among newly diagnosed TB patients, and the roles of food insecurity and HIV status on (a) depression, and (b) anxiety. Patients were categorised as having depression if they scored 10 points or more on PHQ-9^(19)^, and having anxiety if they scored 36 points (raw score) or more on ZUNG^(21)^. Prevalence estimates of depression and anxiety were calculated as the number of observed participants with depression and anxiety, respectively, divided by the total number of enrolled participants, and the 95 % confidence limits were obtained by the Wilson score method. Based on Household Food Insecurity Access Scale scores, participants were categorised as ‘food secure’, ‘mildly insecure’, ‘moderately insecure’ or ‘severely insecure’ households^(22)^. For the purpose of analysis, we dichotomised participants as experiencing ‘food security or mild insecurity’ or ‘moderate to severe insecurity’^(23)^. Monthly income was categorised as having ‘no income’ or ‘some income’. As recommended for estimating prevalence ratios in cross-sectional studies where the outcome of interest was common, Poisson regression models with robust variance were used to examine the bivariate associations between selected covariates and each of the mental disorder outcomes^(24)^. Independent covariates were selected based on *a priori* knowledge of predictors of adverse mental health, which include age, gender, HIV status, monthly income, education, marital status and food insecurity. We excluded education and marital status in the multivariable model due to the relatively small overall sample size of our study. Subsequently, we analysed the bivariate associations between age, gender, monthly income, ART status, CD4 T-cell count, food insecurity and each outcome among participants infected with HIV. Similarly, multivariable model was constructed to determine the independent effect of each covariate, regardless of their bivariate associations. However, because this is a smaller subset of participants, we present the model including only food insecurity, ART status and CD4 T-cell count in this paper. Estimates were similar between the smaller model and the full model.

Data analysis was carried out in SAS version 9.4 (SAS Institute). In accordance with recent guidelines, no alpha cut-off was specified for statistical significance^(25)^.

## Results

Between January and December 2019, 180 participants were recruited into the study. Overall, 99 (55·0 %) were HIV positive, 64 (35·6 %) were female, 155 (86·1 %) were single, 137 (76·1 %) were diagnosed with pulmonary TB, 17 (9·4 %) had no formal education and 65 (36·1 %) had no income (Table 1). Among those who were HIV co-infected, the median duration since HIV diagnosis was 43 months, median CD4 T-cell count was 335 and 31 (31·3 %) had never taken ART, of whom 90 % were newly diagnosed with HIV at or within one month of study enrolment. Over half of all participants reported being food secure, and 15 (8·4 %), 17 (9·5 %) and 52 (29·0 %) reported experiencing mild insecurity, moderate insecurity and severe insecurity, respectively (Table 1).

**Table 1:** Characteristics of study participants in Botswana, 2019 (*n* 180)

Characteristics	HIV positive		HIV negative	
	*n*	%	*n*	%
Gender				
Male	62	62·6	54	66·7
Female	37	37·4	27	33·3
Age				
Median	41		29	
IQR	31–47		23–39	
Marital status				
Married	9	9·1	9	11·1
Single	86	86·9	69	85·2
Divorced/separated/widow	4	4·0	3	3·7
Education				
None	11	11·1	6	7·4
Secondary or less	74	74·7	49	60·5
Tertiary or certificate	7	7·1	12	14·8
Diploma or degree	7	7·1	13	16·1
Masters or PhD	0		1	1·2
Income (in Botswana pula*)				
No income	35	35·4	30	37·0
<5000	53	53·5	45	55·6
5000–10 000	9	9·1	5	6·2
>10 000	2	2·0	1	1·2
TB diagnosis methods				
Culture	0		2	2·5
XPERT	34	34·3	38	46·9
AFB	8	8·1	15	18·5
Chest X-ray	27	27·3	11	13·6
Clinical	30	30·3	15	18·5
TB symptoms length				
None	6	6·1	0	
<1 month	47	47·5	42	51·8
1–2 months	19	19·2	22	27·2
2–3 months	12	12·1	8	9·9
>3 months	15	15·1	9	11·1
Smoking				
No	75	75·8	60	74·1
Yes	24	24·2	21	25·9
Alcohol				
No	57	57·6	44	54·3
Yes	42	42·4	37	45·7
Disease classification†				
Pulmonary	71	72·5	66	81·5
Extrapulmonary	27	27·5	15	18·5
Time since HIV diagnosis (in months)‡				
Median	43		N/A	
IQR	1–119			
CD4 T-cell count at time closest to TB diagnosis (cells/mm^3^)§				
Median	335		N/A	
IQR	120–469			
ART history				
Never taken ART	31	31·3	N/A	
Taking ART	59	59·6		
Took ART but stopped	9	9·1		
Depression				
No depression (0)	3	3·0	5	6·2
Minimal depression (1–4)	27	27·3	20	24·7
Mild depression (5–9)	40	40·4	38	46·9
Moderate depression (10–14)	15	15·2	11	13·6
Moderately severe depression (15–19)	11	11·1	3	3·7
Severe depression (20–27)	3	3·0	4	4·9
Anxiety				
Normal (20–35)	50	50·5	45	55·6
Minimal to moderate anxiety (36–47)	39	39·4	32	39·5
Marked to severe anxiety (48–59)	7	7·1	4	4·9
Most extreme anxiety (≥60)	3	3·0	0	
Depression–Anxiety co-existence				
No depression, no anxiety	44	44·4	41	50·6
Depression, no anxiety	6	6·1	4	4·9
Anxiety, no depression	26	26·3	22	27·2
Depression and anxiety	23	23·2	14	17·3
Household food insecurity				
Secure	51	51·5	45	55·6
Mildly insecure	8	8·1	7	8·6
Moderately insecure	11	11·1	6	7·4
Severely insecure	29	29·3	23	28·4

IQR, interquartile range; TB, tuberculosis; XPERT, GeneXpert MTB/RIF; AFB, acid-fast Bacilli smear; ART, antiretroviral therapy.

* 5000 Botswana pula is approximately 465 US dollars.

† *n* = 1 missing data on disease classification.

‡ *n* = 2 missing data on HIV diagnosis date.

§ *n* = 14 missing data on CD4 T-cell count.

The overall prevalence of depression, anxiety and co-existence of both conditions was 26·1 %, 47·2 % and 20·6 %, respectively. Among those who were depressed, 26 (14·4 %), 14 (7·8 %) and 7 (3·9 %) had moderate, moderately severe and severe depression, respectively (Table 1). Twenty-seven (15·1 %) participants had PHQ-9 scores indicative of suicidal ideation. Additionally, 71 (39·4 %), 11 (6·1 %) and 3 (1·7 %) had minimal to moderate, marked to severe and most extreme anxiety, respectively.

Table 2 shows the results of Poisson regression analysis of covariates associated with depression. In bivariate analysis, moderate to severe food insecurity was associated with a higher prevalence of depression (crude prevalence ratio (PR) = 2·37; 95 % CI 1·44, 3·91). After adjusting for age, gender, monthly income and HIV co-infection status, the association between moderate to severe food insecurity and depression remained similar (adjusted PR = 2·30; 95 % CI 1·40, 3·78).

**Table 2:** Correlates of depression* among study participants in Botswana, 2019 (*n* 180)

Variables	Prevalence		Crude PR	95 % CI	Adjusted PR†	95 % CI
	*n*/*N*	%				
Age	N/A		1·01	0·99, 1·03	1·01	0·99, 1·02
Gender						
Male	26/116	22·4	1·00		1·00	
Female	21/64	32·8	1·46	0·90, 2·38	1·41	0·88, 2·27
HIV						
Negative	18/81	22·2	1·00		1·00	
Positive	29/99	29·3	1·32	0·79, 2·19	1·20	0·73, 1·98
Income						
No	19/65	29·2	1·00		1·00	
Yes	28/115	24·4	0·83	0·51, 1·37	0·95	0·59, 1·52
Education						
No	5/17	29·4	1·00		Not included	
Yes	42/163	25·8	0·88	0·40, 1·91		
Marital status						
Single/divorced or separated/widow	3/18	16·7	1·00		Not included	
Married	44/162	27·2	0·61	0·21, 1·78		
Food insecurity						
None or mildly insecure	19/111	17·1	1·00		1·00	
Moderate to severe insecure	28/69	40·6	2·37	1·44, 3·91	2·30	1·40, 3·78

PR, prevalence ratio.

* The presence of depression is defined as scoring equal to or more than 10 points on the Patient Health Questionnaire-9.

† The multivariable Poisson regression analysis adjusted for age, gender, HIV status, income and food insecurity.

Similar findings were observed for the outcome of anxiety; moderate to severe food insecurity was associated with a higher prevalence of anxiety (crude PR = 1·43; 95 % CI 1·06, 1·93; Table 3). The association remained robust in multivariable Poisson analysis after adjusting for age, gender, monthly income and HIV co-infection status (adjusted PR = 1·41; 95 % CI 1·05, 1·91; Table 3).

**Table 3:** Correlates of anxiety* among study participants in Botswana, 2019 (*n* 180)

Variables	Prevalence		Crude PR	95 % CI	Adjusted PR^†^	95 % CI
	*n/N*	%				
Age	N/A		1·01	0·99, 1·02	1·01	0·99, 1·02
Gender						
Male	52/116	44·8	1·00		1·00	
Female	33/64	51·6	1·15	0·84, 1·57	1·14	0·84, 1·56
HIV						
Negative	36/81	44·4	1·00		1·00	
Positive	49/99	49·5	1·11	0·81, 1·53	1·05	0·77, 1·42
Income						
No	32/65	49·2	1·00		1·00	
Yes	53/115	46·1	0·94	0·69, 1·30	0·98	0·72, 1·34
Education						
No	8/17	47·1	1·00		Not included	
Yes	77/163	47·2	1·00	0·59, 1·70		
Marital status						
Single/divorced or separated/widow	8/18	44·4	1·00		Not included	
Married	77/162	47·5	0·94	0·54, 1·61		
Food insecurity						
None or mildly insecure	45/111	40·5	1·00		1·00	
Moderate to severe insecure	40/69	58·0	1·43	1·06, 1·93	1·41	1·05, 1·91

PR, prevalence ratio.

* The presence of anxiety is defined as scoring equal to or more than 36 points (raw score) on the Zung Anxiety Self-Assessment Scale.

† The multivariable Poisson regression analysis adjusted for age, gender, HIV status, income and food insecurity.

Among HIV-co-infected participants, the estimates were similar in the bivariate analysis for depression (crude PR = 2·41; 95 % CI 1·28, 4·55; Table 4) and anxiety (crude PR = 1·54; 95 % CI 1·04, 2·27). Accounting for ART status and CD4 count closest to the time of TB diagnosis, food insecurity continued to be associated with increased symptoms of depression (adjusted PR = 2·33; 95 % CI 1·24, 4·38) and anxiety (adjusted PR = 1·53; 95 % CI 1·03, 2·26) in the multivariable model.

**Table 4:** Correlates of depression* and anxiety† among HIV-infected participants in Botswana, 2019 (*n* 99)

Variables	Depression				Anxiety			
	Crude PR	95 % CI	Adjusted PR	95 % CI	Crude PR	95 % CI	Adjusted PR	95 % CI
Age	1·01	0·99, 1·04	Not included		1·01	0·99, 1·03	Not included	
Gender								
Male	1·00		Not included		1·00		Not included	
Female	1·56	0·86, 2·86			1·16	0·78, 1·72		
Income								
No	1·00		Not included		1·00		Not included	
Yes	0·67	0·37, 1·23			0·86	0·58, 1·29		
ART								
No	1·00		1·00		1·00		1·00	
Yes	1·29	0·67, 2·47	1·14	0·58, 2·21	1·17	0·77, 1·78	1·12	0·73, 1·73
CD4 T-cell count (cells/mm^3^)								
≤200	1·00		1·00		1·00		1·00	
>200	1·48	0·70, 3·11	1·26	0·59, 2·72	1·17	0·72, 1·91	1·06	0·64, 1·75
Missing	0·92	0·28, 3·03	0·87	0·26, 2·94	1·32	0·72, 2·43	1·27	0·68, 2·35
Food insecurity								
None or mildly insecure	1·00		1·00		1·00		1·00	
Moderate to severe insecure	2·41	1·28, 4·55	2·33	1·24, 4·38	1·54	1·04, 2·27	1·53	1·03, 2·26

PR, prevalence ratio; ART, antiretroviral therapy.

* The presence of depression is defined as scoring equal to or more than 10 points on the Patient Health Questionnaire-9.

† The presence of anxiety is defined as scoring equal to or more than 36 points (raw score) on the Zung Anxiety Self-Assessment Scale.

## Discussion

In our population of TB patients, we showed that household food insecurity was the most important factor associated with symptoms of depression and anxiety, independent of HIV co-infection status, age, monthly income and gender. Our findings confirmed previous research that showed a linkage between food insecurity and poor mental health^(26,27)^ and extended this finding in TB patients. Several reasons may explain this association. First, as a consequence of food insecurity, deficiency in critical micronutrient could lead to depression and anxiety via biological pathways^(28)^. Additionally, food insecurity encompasses not only hunger and under-nutrition but also uncertainty and distress over the access of food. Thus, food insecurity may compromise mental health by generating instability and unpredictability into lives of the already socio-economically vulnerable TB population^(26)^. This pathway could be independent of the degree of social and economic deprivation, as a global analysis of 149 countries concluded an association between food insecurity and mental illness in a dose-response fashion, after controlling for socio-economic status^(27)^.

To our knowledge, this is the first study that examined the association between food insecurity and mental illness among new TB patients in a high burden setting. In compliance with The End TB Strategy, Botswana has social (e.g. food support) programmes to help TB-affected individuals to improve nutritional status and treatment adherence and alleviate the steep costs associated with the disease and treatment. Specifically, TB patients with confirmed diagnosis may receive food allocations that include cooking oil, beans and maize flour with additive vitamins. However, the food rations are usually not enough to support every member of the household. In addition, TB renders many patients unable to work and contribute to household expenditures, and caregivers may still experience household food insecurity as they face challenges of providing for their family due to income loss^(29)^. The total financial costs of TB can be catastrophic for patients – up to 58 % of annual individual income and 39 % of household income in low- and middle-income countries, and income loss constitutes the largest portion of all TB-related cost at 60 %^(29)^. The cyclical nature of the relationship between food insecurity and TB fosters sustained impoverishment and makes it difficult for household to break the cycle without outside intervention. Malnutrition, a consequence of food insecurity, may predispose patients for risks of TB infection and TB disease reactivation^(30)^.

Comparable with national data released by FAO^(17)^, our survey indicated 38·5 % of the participants had experienced moderate to severe food insecurity prior to receiving their TB diagnosis. Further, a separate survey estimated that only 12 % of households report to be ‘food secure’ in urban parts of Gaborone^(16)^. These findings highlight the pervasive food-insecure households among TB patients and those at risk for TB in Botswana, which lend support for food assistance programmes and social protection interventions to target populations who are at high risks for TB. Other research suggests members of households who participate in food supplementation programmes have lower rates of psychological distress; by reducing food insecurity, these programmes can be effective in improving mental health and overall security among vulnerable populations^(31)^.

In this study, we revealed that anxiety and depression were highly prevalent among patients newly diagnosed with TB in Botswana. Approximately 26 % of enrolled TB patients had PHQ-9 scores indicative of moderate to severe depression and 15 % indicative of suicidal ideation. Our estimate was comparable to recent studies in several countries that examined depression (moderate to severe) among TB patients, including Ethiopia (17·6–54·0 %)^(32–34)^, South Africa (32·9 %)^(13)^, Angola (49·4 %)^(35)^ and China (18·1 %)^(36)^. They were, however, considerably lower than those found in Ethiopia using PHQ-9 by Ambaw *et al.* ^(32)^; these differences may have arisen from variations in study location and population. For example, roughly 45 % of the participants in the Ethiopian study had no formal education and over half were married.

Approximately 46 % of our participants reported anxiety symptoms, comparable with published estimates of other settings: Ethiopia (41·5 %)^(34)^, Angola (38·3 %)^(35)^ and China (18·4 %)^(36)^. None of those studies utilised the ZUNG scale; instead, Generalized Anxiety Disorder Questionnaire and Hospital Anxiety and Depression Scale were used to assess the presence of anxiety symptoms, which might be an explanation for the discrepant results. Our estimate was much higher than those published by Wang *et al.* of Chinese TB patients^(36)^. One explanation for this discrepancy could be patient characteristics: in the Chinese study, over half of the participants had tertiary or higher education levels; majority were married and patients with extrapulmonary TB were excluded.

We did not find an association between HIV co-morbidity and mental disorders in our sample. This finding is in agreement with some studies^(32,33)^, but not others^(13,34)^. Studies that reported an association between co-morbidity and mental disorders have posited HIV-associated stigma as the plausible driving force^(13,34)^. Among people with TB, factors such as malnutrition, poor physical health, socio-economic adversities and interaction between these factors may have a stronger influence on depression and anxiety than HIV-related stigma. Furthermore, following WHO’s guidelines in 2015, nearly all sub-Saharan African countries adopted universal HIV care policies irrespective of CD4 T-cell count (‘treat all’), including Botswana. The well-developed, universal access to HIV care may have also influenced mental health in our population of TB patients. Individuals who were co-infected but not on ART predominantly discovered their HIV status at enrolment or within 1 month of study enrolment, and further research is needed among this group as HIV-related stigma may be perceived differently in these individuals.

We note several limitations associated with our research. First, depression and anxiety assessment relied on self-reporting using screening questionnaires. This may lead to self-report bias, particularly under-reporting owing to stigmatisation of psychological disorders. However, we tried to minimise this by training the research staff on administering the questionnaires with consistency and without judgement, and by asking the participants to record their answer on the tablet directly. Second, the PHQ-9 and ZUNG screening tools have not been validated in Botswana, nor in TB patients in Botswana. The English version of these questionnaires was translated by a native speaker of Setswana. Some items on the questionnaires overlap with TB symptoms, including fatigue, loss of appetite and insomnia, which may lead to misclassification of depressive and anxiety cases. As a consequence, our estimate of associations between depression, anxiety and independent variables may be biased towards the null. Third, due to the cross-sectional study design, statistical associations may not be representative of casual relationships between exposure and outcome variables. Finally, confounding by unmeasured variables is a possibility in our study.

## Conclusion

In conclusion, we have shown that common mental disorders are highly prevalent and food insecurity is associated with poor mental health among new TB patients. This study highlights the critical need for integration of TB, mental health and social services (i.e. food assistance) to address the TB-mental health co-morbidity in Botswana. Mental illness is increasingly being recognised as an important driver of the TB epidemic and negatively impacts effective TB control^(2)^. Individually, poor mental health is associated with elevated morbidity and mortality, and greater risk of drug resistance^(3)^. Despite the need, mental health counselling and psychiatric services are severely underfunded in low-resource settings where numerous public health priorities compete^(6)^. Social assistance programmes that mitigate costs of TB care and improve food access may ameliorate symptoms of common mental disorders. Current programmes intended to support individuals struggling with food security may require additional measures to identify patients who experience moderate to severe food insecurity and ensure their access to and adequacy of nutritious and sufficient food for the household. Given the global goal of ending TB and rising interest in mental health, longitudinal research is needed to determine the temporal relationship between food insecurity and mental disorders and to identify potential pathways through which food insecurity may elevate the risk of common mental disorders among TB patients.
